# Nanoemulsion and Nanoliposome Based Strategies for Improving Anthocyanin Stability and Bioavailability

**DOI:** 10.3390/nu11051052

**Published:** 2019-05-10

**Authors:** Bing-Huei Chen, Baskaran Stephen Inbaraj

**Affiliations:** Department of Food Science, Fu Jen Catholic University, New Taipei City 242, Taiwan; sinbaraj@yahoo.com

**Keywords:** anthocyanin, physicochemical stability, nanotechnology, nanoemulsion, nanoliposome, bioavailability, biological activity

## Abstract

Background: Anthocyanins, a flavonoid class of water-soluble pigments, are reported to possess several biological activities, including antioxidant, anti-inflammatory, and anti-cancer. However, anthocyanins are highly susceptible to degradation in high pH, light, heat, and oxygen during processing and storage. Conventional microencapsulation techniques fail to provide stability to anthocyanins under physiological environments mainly because of their large particle size as well as low zeta potential and encapsulation efficiency. Methods: Nanotechnology provides novel strategies for preparing nanoformulations to enhance the physicochemical stability of anthocyanins. Nanoemulsion and nanoliposome are the two most commonly used nanosystems in pharmaceutical and food-related fields. In this review, an overview of various nanoemulsion and nanoliposome systems reported recently for enhancing stability, bioavailability, and bioactivity of anthocyanins is presented. Results: Anthocyanin nanoemulsions with different oil, water, surfactant, and cosurfactant ratios were prepared from extracts of mangosteen peel, purple sweet potato, cranberry, red cabbage, blueberry, jaboticaba peel, and acai berry and evaluated for their antioxidant activity, enhancement of physicochemical stability, topical skin application, and urinary tract infection. Likewise, unilamellar and multilamellar nanoliposomes were prepared using different types and levels of lecithin without or with cholesterol from anthocyanin standards and extracts of *Hibiscus sabdariffa*, mulberry, elderberry, black carrot, and pistachio green hull for the evaluation of physicochemical and oxidative stability, in vitro bioaccessibility, and melanogenic activity, as well as protective effects against diabetes mellitus and cataract. Conclusion: This review provides an insight into the current nanotechnology updates on enhancement of anthocyanin stability and biological activity.

## 1. Introduction

Anthocyanins (ANCs) are a group of water-soluble pigments belonging to a flavonoid class of secondary metabolites synthesized by plants [1]. They derive the name from two Greek words, “anthos = flower” and “kianos = blue”, and possess attractive colors ranging from red to magenta, purple, and blue in flower, fruits, and vegetables [2,3]. The ANCs occur primarily as glycosides or acylglycosides of their corresponding aglycones, and over 600 naturally occurring anthocyanins have been reported thus far, varying in (1) number and position of hydroxyl and methoxyl groups, (2) type, number, and position of sugar attachment, and (3) both the type and the extent of sugar acylation [4,5]. Owing to their intense color, the ANCs are regarded as safe natural colorants to replace artificial pigments in the food industry [6]. Additionally, based on many cell models, animal models, and clinical trials, the ANCs were shown to possess antioxidant, anti-inflammatory, and anti-cancer activities, as well as protection against cardiovascular disease, obesity, and diabetes [3,5,7]. However, they are highly susceptible to degradation in high pH, light, heat, and oxygen during processing and storage as well as interaction with other food components and additives, resulting in poor bioavailability and reduced bioactivity [8,9].

Some recent reports indicated that the poor bioavailability of ANCs (<1–2%) could be due to underestimation of biologically active phase I and phase II metabolites, conjugated products, and microbe-generated metabolites [10,11,12]. Also, the presence of carbinol and chalcone forms of ANCs at neutral pH and their inability in returning from chemically-bound states to flavylium cations upon acidification can contribute to low bioavailability [4]. Conventional methods to enhance the stability of ANCs can be through encapsulation with various natural polymers as well as food-based proteins and polysaccharides using several classical microencapsulation techniques that are well documented [13,14,15,16,17]. However, these micro-delivery systems are often unstable in the physiological environment due to their large particle size as well as low zeta potential (ZP) and encapsulation efficiency (EE) [18,19,20,21,22,23]. As the ANCs are hydrophilic, many studies have also focused on encapsulating hydrophilic ANCs into a double-emulsion system (water-in-oil-in-water, W/O/W) which is composed of ANCs in inner water droplets dispersed in large oil droplets, which are further dispersed in an aqueous continuous phase [24,25]. Nevertheless, the W/O/W emulsions are mostly large in particle size and are highly prone to environmental stress, resulting in instability due to flocculation, coalescence, and Ostwald ripening [26]. Also, the encapsulated ANCs can undergo diffusion from inner to outer aqueous phases or oil phases, altering the release pattern and the targeting of ANCs [19]. Thus, it is vital to develop more stable and efficient delivery systems for highly unstable ANCs.

The recent developments in nanotechnology offer several feasible approaches for preparing nanoformulations to enhance physicochemical stability, bioavailability, and biological activity through active or passive targeting [27,28,29,30]. Among various nanosystems, the nanoemulsion and the nanoliposome are the two most commonly used nanoformulations in both pharmaceutical and food-related fields. Because of their large surface area-to-volume ratio, nanoemulsions can provide a higher stability against gravitational separation and aggregation with their physicochemical and biological properties different from the conventional emulsions [31,32,33]. Likewise, nanoliposome involves preparation of conventional liposome first, followed by reducing the particle size using high pressure homogenization, ultrasound, or membrane extrusion [34,35,36]. Compared to micron-sized emulsions and liposome, their nano-forms can greatly enhance stability, bioavailability, and controlled release [28,29]. While most studies deal with encapsulated ANCs prepared by conventional methods, their nano-based systems are less explored. This review aims to overview the recent reports on nanoemulsion and nanoliposome-based preparations used for enhancing stability, bioavailability, and bioactivity of ANCs.

## 2. Biosynthesis of Anthocyanins

**Figure 1:**
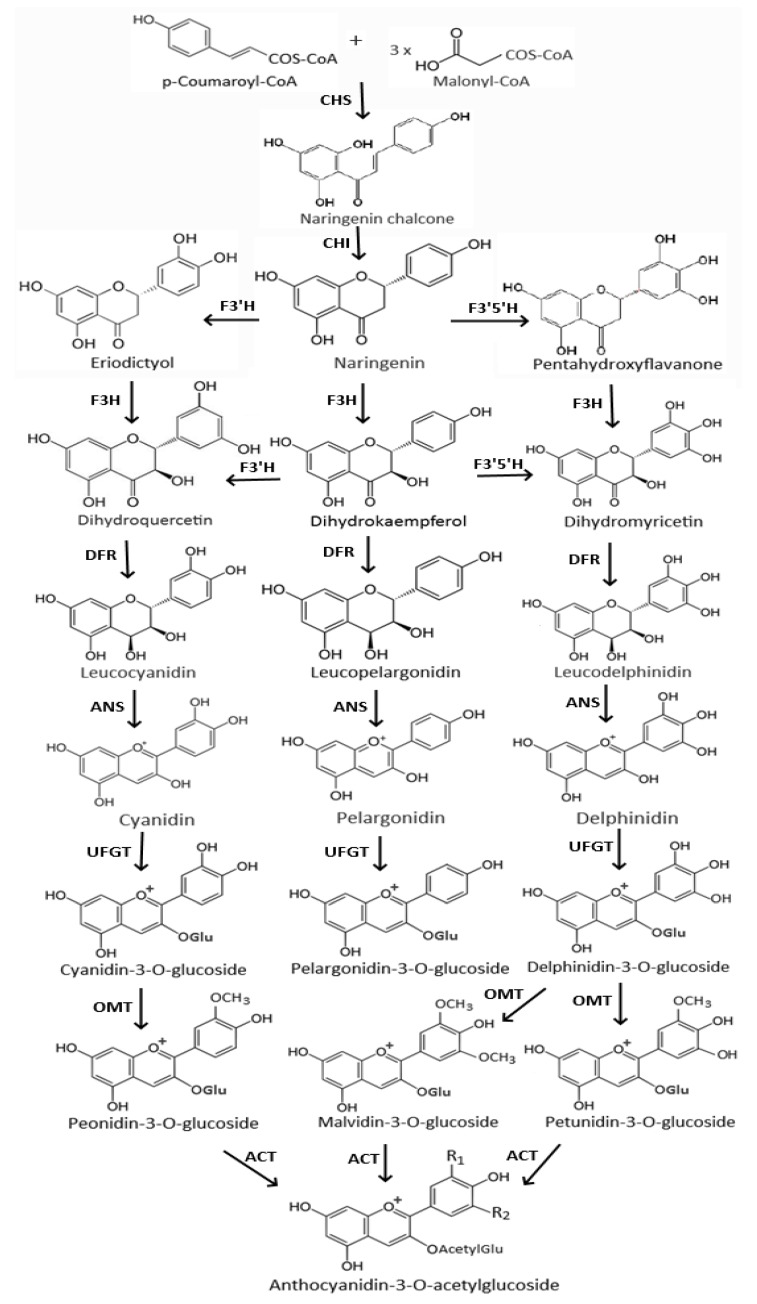
Biosynthesis pathway for anthocyanin production in plants. CHS, chalcone synthase; CHI, chalcone isomerase; F3H, flavanone 3-hydroxylase; F3′H, flavonoid 3′-hydroxylase; F3′5′H, flavonoid 3′,5′-hydroxylase; DFR, dihydroflavonol 4-reductase; ANS, anthocyanidin synthase; UFGT, uridine diphosphate-sugar flavonoid 3-O-glucosyltransferase; OMT, O-methyltransferase; ACT, anthocyanin acyltransferase. The substitution of R_1_ and R_2_ for specific acylated anthocyanins can be referred to chemical structures for all the six common anthocyanidins shown in Figure 2 (adapted with permission from the reference [38]).

Figure 1 shows the biosynthesis pathway of anthocyanins in plants. It is an extension of a general flavonoid pathway, which is initiated through chalcone synthase (CHS)-mediated reaction of one molecule of p-coumaroyl coenzyme A (p-coumaroyl-CoA) with three molecules of malonyl coenzyme A (malonyl-CoA) to form naringenin chalcone [37]. Then, an isomerization of naringenin chalcone in the presence of chalcone isomerase (CHI) yields naringenin, which is eventually converted into eriodictyol and pentahydroxyflavanone by flavanone 3′-hydroxylase (F3′H) and flavanone 3′,5′-hydroxylase (F3′5′H), respectively [38]. Through further hydroxylation by flavonoid 3-hydroxylase (F3H), dihydroquercetin, dihydrokaempferol, and dihydromyricetin are produced, all of which can then undergo reduction to generate their corresponding colorless leucoanthocyanidins by dihydroflavonol-4-reductase (DFR) followed by colored anthocyanidins by anthocyanidin synthase (ANS) [39]. The anthocyanidins are further modified to generate different forms of anthocyanins through glycosylation by uridine diphosphate-sugar flavonoid 3-O-glycosyltransferase (UFGT) and O-methyl transferase (OMT) or acylation by anthocyanin acyltransferase (ACT) [38]. Although the CHS is the key enzyme for initiation of anthocyanin biosynthesis, the anthocyanin composition, the B-ring hydroxylation pattern, the pigmentation, and eventually the color are largely determined by the primary enzymes F3′H and F3′5′H as well as DFR [39,40].

## 3. Chemical Structure and Distribution of Anthocyanins

**Figure 2:**
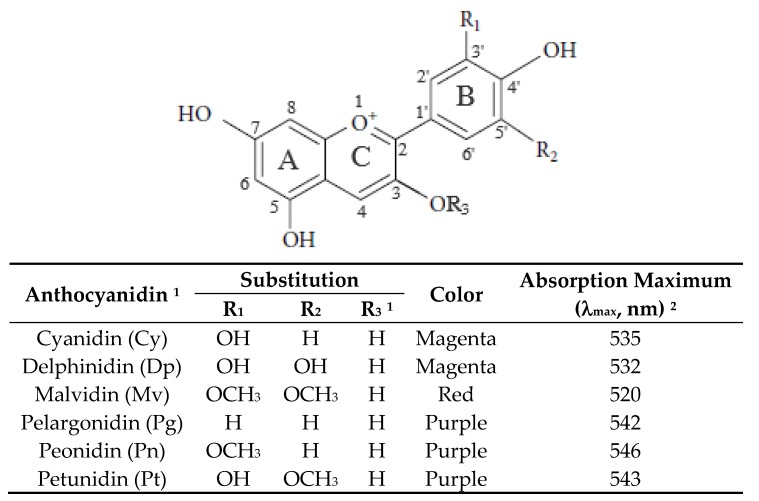
Chemical structure of most common anthocyanins found in fruits and vegetables. Notes: ^1^ the corresponding anthocyanin compounds contain one or two sugar moiety at R_3_ position with glucose-substituted anthocyanidins being the most common anthocyanins found in fruits and vegetables. Sugars can also be present in ring A, and, furthermore, anthocyanins with acylation of sugars with aliphatic and/or aromatic acids can be found. ^2^ The absorption maximum obtained in HCl acidified methanol.

Anthocyanins are substituted glycosides of phenyl-2-benzopyrilium salts (anthocyanidins). Structurally, the anthocyanidins consist of 15 carbon atoms with a typical C6-C3-C6 structural backbone (flavylium cation) containing an aromatic ring (A) bonded to a heterocyclic ring (C) with oxygen at position one, which in turn is connected by a carbon-carbon bond to a third aromatic ring (B) (Figure 2) [8,9]. Two maximum absorption wavelengths occur for anthocyanins, with one between 465 and 550 nm in the visible range and the other between 270 and 280 nm in the UV range [3,41]. Depending on the number and the position of hydroxyl and/or methoxy groups, more than 30 anthocyanidins are found in nature, of which only six—namely, cyanidin (Cy), delphinidin (Dp), pelargonidin (Pg), peonidin (Pn), petunidin (Pt), and malvidin (Mv)—are widely distributed [1,42]. Unlike flavonoids, a long chromophore of eight conjugated double bonds with a positive charge on oxygen in the heterocyclic C-ring is responsible for the intense color of anthocyanins under acidic conditions. The color of these aglycones varies with a high number of hydroxyl groups contributing to a blue color, while a high number of methoxy groups yield a red color [9]. The structural diversity also increases through glycosylation of anthocyanidins with one or more sugars and acylation of sugar molecules with organic acids through ester bonds [43]. Both glycosylation and acylation can modify the molecular size and the polarity of anthocyanins and can eventually alter their physico-chemical properties. For instance, glycosylation increases water solubility, while acylation decreases water solubility [3].

Anthocyanins are distributed in vacuoles of many plant tissues, and both the composition and the total anthocyanin content vary substantially among different plant species and cultivars [6]. They are accumulated largely in flowers and fruits, followed by leaves, stems, and storage organs [2]. Andersen and Jordheim [1] estimated the abundance of six common anthocyanidins (Cy, Dp, Pg, Pn, Mv and Pt) to be 30%, 22%, 18%, 7.5%, 7.5%, and 5%, respectively. The three non-methylated anthocyanidins (Cy, Dp, and Pg) are the most common in nature, contributing to 80% in pigmented leaves, 69% in fruits, and 50% in flowers [44]. Among numerous glycoside derivatives, 3-monosides, 3-biosides, 3,5-biosides, and 3,7-glucoside derivatives are more common, with Cy-3-glucoside (Cy3G) being the frequently occurring anthocyanin in nature [45]. A wide-range content of anthocyanins was reported in edible plants with berries providing the most anthocyanins per serving. Wu et al. [6] determined the anthocyanin content in several common fruits and vegetables in the United States and found chokeberry to contain the highest level (1480 mg/100 g fresh weight) and gooseberry to contain the lowest level (0.7 mg/100 g fresh weight) (Table 1). Moreover, the overall daily consumption of anthocyanins was estimated to be 12.5 mg/person [6].

## 4. Stability of Anthocyanins

The anthocyanins are highly unstable and susceptible to degradation with several factors affecting their stability, including chemical structure, concentration, solvents, pH, storage temperature, light, and oxygen, as well as the presence of enzymes, metallic ions, proteins, and flavonoids. For stability in solvent, Ito et al. [46] demonstrated synthetic flavylium salt solution in protic and aprotic solvents respectively exhibiting red and yellow colors due to the formation of a monomer and a dimer. Accordingly, the red color is favored at high flavylium concentration and high water proportion in organic solvents. The pH is an important factor affecting anthocyanin stability. The anthocyanins exist in the form of flavylium cations at highly acidic conditions (red color at pH < 2). Following a rise in pH, the cation form undergoes hydration to form colorless carbinol pseudo base with hemiacetal structure through a nucleophilic attack of water in the C2 position and subsequently colorless chalcone is formed slowly through C-ring opening [8,43]. Upon increasing the pH above five, the flavylium cation undergoes deprotonation to generate a neutral quinonoidal base (purple color) at slightly acidic to neutral conditions. Accordingly, the stability can be defined as the inability of flavylium cations to convert into colorless carbinol pseudobases and chalcone forms [8]. The higher the conversion inability is, the higher the stability is, which is a significant phenomenon from the perspective of food application. For example, the anthocyanin stability declines with C5 substitution, while the stability slightly rises upon glycosylation at C3 position [47,48]. However, the acylation with organic acids (especially phenolic acids) substantially increases the anthocyanin stability by steric hindrance, with the extent of anthocyanin stability being significantly affected by both the type and the degree of acylation [3,8]. On the contrary, the presence of sulfite, sulfur dioxide, ascorbic acid, amino acid, phenols, sugar derivatives, and some enzymes can promote anthocyanin decoloration through various mechanisms [8].

Copigmentation is a unique phenomenon involved in stabilization of anthocyanins through association mechanisms such as self-association, intramolecular, and intermolecular copigmentation. It causes vertical stacking of anthocyanidins and copigments accompanied by a bathochromic shift and a hyperchromic effect (enhancement of color intensity) [49]. The advantage of copigmentation is that a wide range of different type of compounds, such as phenolic acids, flavonoids, amino acids, alkaloids, purines, polysaccharides, and anthocyanin, can be used as copigments [8]. However, the copigmentation efficiency can depend on type and concentration of both anthocyanin and copigment as well as temperature, pH, and solvent type. Anthocyanins with o-dihydroxy groups (vicinal hydroxyl groups) in the B-ring are stabilized by conjugating with several metal ions including Fe^3+^, Al^3+^, Mg^2+^, Sn^2+^, Cu^2+^, and Mo^2+^, which is accompanied by a bathochromic shift resulting in a blue color [8,44]. Stabilization of anthocyanin through the formation of pyranoanthocyanins has been reported in red wines, black carrot juice, and orange juice by cyclization between C4 and hydroxyl group in C5 position of flavylium cations, generating a new fourth ring upon the reaction with molecules such as 4-vinylphenol, acetaldehyde, pyruvic acid, flavonols, and organic acids [44,50,51]. Some recent studies have demonstrated the enhancement of anthocyanin stability through interaction with polymeric hydrocolloids and metal ions in processed foods [43,52,53]. Based on this approach and recent developments in the field of nanotechnology, the stability of anthocyanin can be substantially improved through the encapsulation of anthocyanin into nanoformulations such as nanoemulsions and nanoliposomes.

## 5. Nanoemulsion-Based Stability and Bioavailability of Anthocyanins

Nanoemulsions are kinetically stable and thermodynamically unstable colloidal systems formed by mixing oil, emulsifier, and water [31]. They are optically transparent or semi-transparent, and their particle size can be significantly affected by the type and the ratio of components as well as the mechanical and the shearing forces [26,32]. Two types of nanoemulsions can be prepared, namely, oil-in-water (O/W) and water-in-oil (W/O), with the former being most commonly used. Nanoemulsions are usually prepared by either low-energy (LEM) or high-energy (HEM) methods or a combination of both [31]. The LEMs involve spontaneous emulsification and phase inversion techniques to obtain nano-sized particles through modifying the composition of surfactant-oil-water mixtures or temperature [32,33]. On the other hand, the HEMs use mechanical devices such as high pressure homogenizers, microfluidizers, or ultrasonic homogenizers to generate intense disruptive forces for reducing the particle size. The main advantages of LEMs is that they are simple, fast, and less expensive than HEMs, while HEMs require low surfactant levels, and a wide range of ingredients can be used [32,33]. The nanoemulsions can be tailored for specific applications by controlling the particle size distribution and the concentration as well as characteristics of interfacial layers such as thickness, composition, and electrical properties [26]. An overview of several nanoemulsions reported for enhancing anthocyanin stability and bioavailability is presented in the following section.

**Figure 3:**
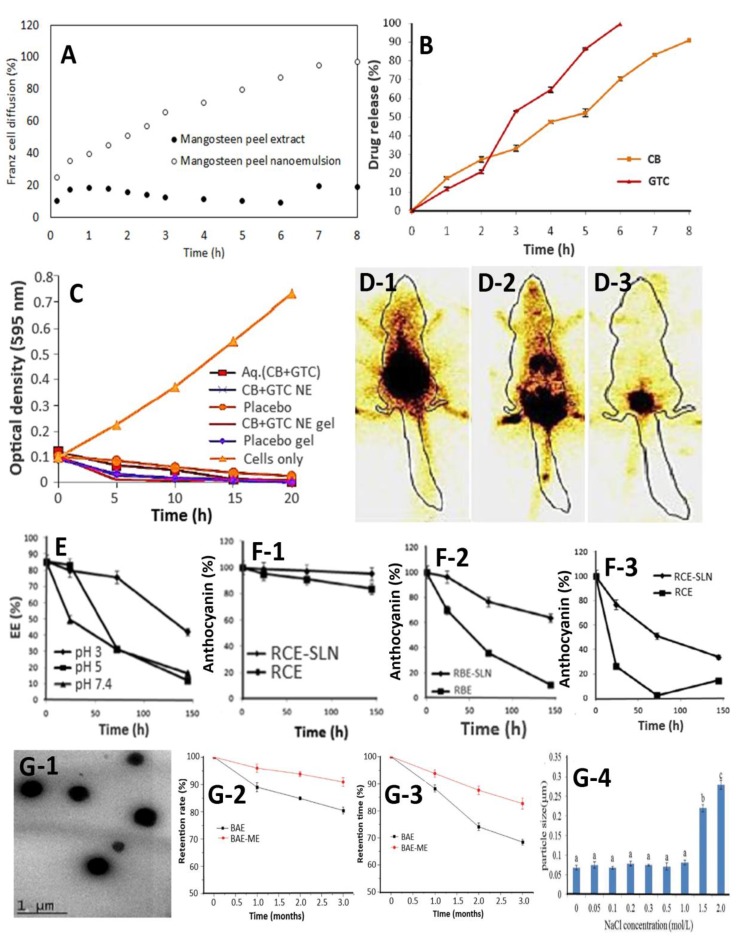
In vitro and in vivo evaluation of anthocyanin nanoemulsion prepared from several natural fruit/vegetable extracts. Panel (**A**), Anthocyanin nanoemulsion from mangosteen peel showing higher diffusion in in vitro Franz cell diffusion test than extract (topical skin application). Panel (**B**), cranberry and green tea catechin extracts nanoemulsion gel (CB + GTC NE gel) showing high in vitro release of CB and GTC into simulated vaginal fluid. Panel (**C**), CB + GTC NE gel showing faster growth inhibition of *Escherichia coli* compared to other treatments. Panel (**D1–3**), Gamma scintigraphy images of female Sprague-Dawley rats after oral (**D1**) and intravaginal (**D2**) administration of radiolabeled CB+GTC NE gel (^99m^Tc-CB/GTC-NE gel) as well as intravaginal administration of radiolabeled CB+GTC aqueous mixture (^99m^Tc-Aq.CB+GTC) (**D3**). Panels (**E**) and (**F1–3**), stability of red cabbage anthocyanin-based solid-lipid nanoparticles (RCE-SLN) as affected by different pH (E) and temperatures at 25 °C (F1), 45 °C (F2), and 60 °C (F3) during storage for six days. Panel G1-4, TEM image (G1) of anthocyanin microemulsion prepared from blueberry extract (BAE-ME) along with its stability as affected by storage over a three month period at 4 °C (G2) and 25 °C (G3) as well as different ionic strengths (G4) (adapted with permission from references [54,58,59,60]).

Pratiwi et al. [54] prepared a nanoemulsion with anthocyanin-rich mangosteen peel extract (MPE-NE) as a raw material, aiming to develop a self-nanoemulsifying drug delivery system (SNEDDS) by a simplex lattice design method. Through incorporation of the ethyl acetate extract, an optimum SNEDDS was obtained with virgin coconut oil, Tween 80, and polyethylene glycol 400 (PEG 400) at a ratio of 1:6.95:2.05. This SNEDDS showed a much higher diffusion (97%) within 8 h in an in vitro Franz diffusion permeation model compared to the unloaded-SNEDDS (19%) (Figure 3A), suggesting that the SNEDDS formulation with MPE can increase penetration of predominant α-mangostin through *stratum corneum*. Interestingly, a clear transparent SNEDDS with transmittance at 92% was obtained within a short time (65 s) and was physically stable for three months, as evidenced by the particle size, the ZP, and the drug loading being 20 nm, −12.40 mV, and 125 mg/5 mL, respectively. More recently, a nanoemulsion with ethyl acetate MPE as a raw material was prepared by Mulia et al. [55] using a high-speed homogenization method. A stable MPE-NE with a particle size of 181 nm, a ZP of −30.9 mV, and an α-mangostin level at 0.01% could be obtained at an optimum homogenizer speed at 8000 rpm and a virgin coconut oil/Tween 80/Span 80 surfactant (hydrophilic-lipophilic balance, HLB = 12) volume ratio at 1:1.4. The MPE-NE was physically stable for 28 days without phase separation, and the accelerated stability test revealed a shelf-life stability of one year. The above two studies mainly focused on stability issues, suggesting the obtained MPE-NE may be used for topical application.

Additionally, in two different studies, Desnita et al. [56,57] prepared a W/O microemulsion with an anthocyanin-rich ethanolic extract from purple sweet potato extract (SPE-ME) for topical skin application by varying surfactant/cosurfactant type and level. In the first study, an aqueous phase consisting of SPE and dimethylol dimethyl-hydantoin was added to an oil phase composed of Span 60 (0.75, 1 and 1.25%)/PEG 400 at 1:1 ratio, butylated hydroxytoluene (BHT) (0.1%), and olive oil, which was followed by stirring at 1000 rpm for 90 min at 40 °C and sonicating for 24 min to obtain a clear and transparent SPE-ME. However, the oil phase containing Span 60/PEG 400 was changed to only Span 80 (20, 25, and 30%) in the second study with the remaining components and preparation procedure being unchanged. Through evaluation of physical stability for 28 days, the most stable SPE-ME could be obtained by using 0.75% Span60/PEG 400 (1:1) or 20% Span 80 with pH at 5.8–5.9 (safe for skin) with the particle size at 111.1–152.4 nm. For evaluation of antioxidant activity by 1,1-diphenyl-2-picrylhydrazyl (DPPH) assay, the SPE-ME obtained by both studies showed the same half-maximal inhibitory concentration (IC_50_) value of 38.25 μg/mL with 81% inhibition of free radicals [56,57].

As both anthocyanin-rich cranberry (CB) and catechin-rich green tea (GTC) are well known for their high anti-microbial activity, Kaur et al. [58] developed a nanoemulsion-based gel from cranberry powder and polyphenol 90 (commercial GTC) for treatment of urinary tract infection. The CB/GTC-NE with a particle size at 58 nm, a polydispersity index (PDI) at 0.2, and a ZP at −16 mV was successfully prepared by a formula containing 5% oil (oleic acid), 16.4% emulsifier (Tween 20+glycerol), and 41 mg/mL total drug content (30 mg/mL CB+11 mg/mL GTC) and subjected to high shear homogenization (10,000 rpm for 30 min) and high energy ultrasonication (300 s at 30% amplitude) followed by mixing with 1% chitosan in 1% lactic acid for preparation of a CB/GTC-NE gel. A high level of in vitro release of CB (99.4%) and GTC (90.9%) into simulated vaginal fluid (Figure 3B) as well as a faster growth inhibition of *Escherichia coli* was shown by treatment with CB/GTC-NE gel for 5 h (Figure 3C). Intravaginal administration of radiolabeled metastable Technetium-99 isomer radiolabeled CB/GTC-NE (^99m^Tc-CB/GTC-NE) gel to female Sprague-Dawley (SD) rats during gamma scintigraphy study further revealed the gel transport from the vaginal cavity into the systemic circulation (Figure 3D1-3), accompanied by a significant uptake by the kidney (3.20%/g gel) and the urinary bladder (3.64%/g gel) [58].

To enhance the stability of anthocyanins from red cabbage under gastrointestinal (GI) conditions, Ravanfar et al. [59] incorporated the red cabbage extract into solid lipid nanoparticles (SLNs) through dilution of W/O microemulsion. The aqueous phase containing red cabbage anthocyanin extract (RCE) was titrated against the lipid phase prepared by heating palmitic acid and surfactants (Span 85 and egg lecithin) at 60 °C, followed by adding ethanol as cosurfactant (surfactant-cosurfactant = 1:1), stirring at 800 rpm, and finally dispersing in an aqueous pluronic F127 stabilizer. By optimization of the SLN formulation parameters using a pseudo ternary phase diagram as well as a Placket-Burman and a Box-Behnken experimental design, the spherical-shaped RCE-SLNs with a particle size of 455 nm and a high EE of 89.2% could be obtained using 10% volume of the primary aqueous phase in the lipid phase, 50% of the total surfactant, and 0.1% volume of the lipid phase to the secondary aqueous phase. The RCE-SLNs possessed a higher stability at pH 3.0 (gastric fluid) than at pH 5.0 (intestinal fluid) (Figure 3E), while a low-temperature storage (<25 °C) could protect anthocyanins from degradation (Figure 3F) [59].

**Figure 4:**
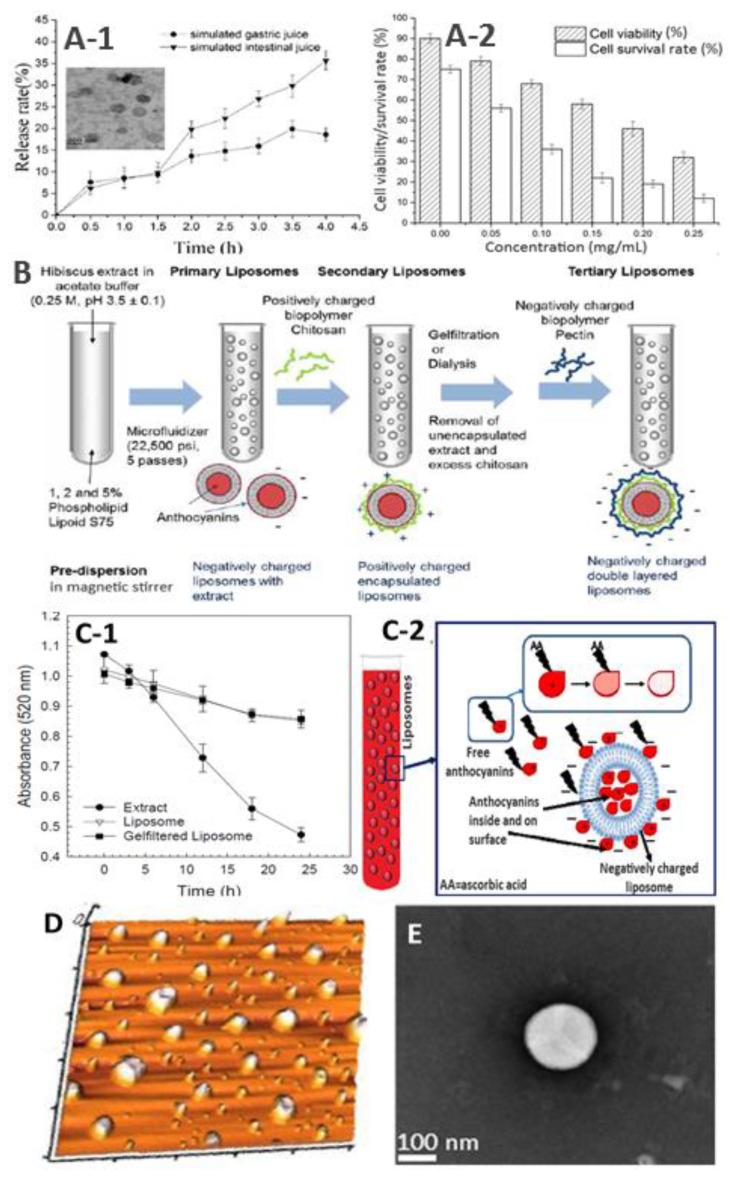
Different panels showing in vitro release rate (**A1**) and absorption efficiency using the Caco-2 cell model (**A2**) with the inset in A1 showing TEM image of cyanidin-3-glucoside nanoliposome, schematic representation of multilayered anthocyanin nanoliposome prepared from hibiscus (**B**), the physical stability of black carrot anthocyanin nanoliposome and extract in the presence of ascorbic acid (**C-1**) along with the proposed mechanism of anthocyanin protection (**C-2**), and atomic force microscopy (AFM) (**D**) and TEM (**E**) images of anthocyanin nanoliposomes prepared from pistachio hull and bilberry extracts, respectively (adapted with permission from references [65,68,69,70,71]).

In a similar study, blueberry anthocyanins (BAE) were incorporated into a microemulsion system (BAE-ME) for encapsulation by Chen et al. [60]. Through development of a pseudo ternary phase diagram composed of isopropyl myristate, Tween 80/Span 80 and ethanol as oil, surfactant, and cosurfactant, respectively, a stable W/O BAE-ME system with a particle size at 70 nm and anthocyanin content at 425.5 μg/g was prepared using an optimum oil phase-to-emulsifier ratio at 7:3, a surfactant-to-cosurfactant ratio at 2:1, and a hydrophilic-lipophilic balance (HLB) value at 7.5 (Figure 3G-1). Storage at different temperatures (4 °C, 25 °C, and 60 °C for 10 days) and light conditions (natural and ultraviolet light for 25 days) was shown to enhance anthocyanin degradation with increasing storage time. However, the anthocyanins incorporated into ME for encapsulation showed higher retention than free anthocyanins at both 4 °C (Figure 3G-2) and 25 °C (Figure 3G-3). Also, the BAE-ME remained stable to the ionic strength at ≤1.0 mol/L NaCl (Figure 3G-4) and sugar level (glucose-sucrose combination) up to 9%. In another study, Bamba et al. [61] used whey protein isolate (WPI) and anthocyanin-rich blueberry pomace extract to prepare a W_1_/O/W_2_ double emulsion system (BPAE-NE) and evaluated the optimum homogenization condition. The BAE obtained by ultrasonic-assisted extraction with 50% ethanol (40 °C for 60 min) was added drop-wise to the oil phase containing corn oil and polyglycerol polyricinoleate, followed by homogenizing for 10 min to obtain a W_1_/O emulsion, which was then mixed with the WPI solution in 0.02% sodium benzoate and passed once through a high pressure homogenizer to obtain a W_1_/O/W_2_ double emulsion. Through evaluation of different homogenization pressures (50–200 MPa), speeds (6000–12000 rpm), and times (15–20 min), a stable W_1_/O/W_2_ double emulsion system with an average particle size at <400 nm, PDI at <0.25, and ZP at <(−40 mV) was obtained at 50 MPa, 6000 rpm, and 10 min. Nevertheless, the stability of this W_1_/O/W_2_ double emulsion remained unexplored.

Extracts from Jaboticaba peel (*Myrciaria cauliflora*) containing 2.56% of total flavonoids and 0.80% of anthocyanins were incorporated into the nanoemulsion (JPE-NE) prepared by mixing the extract and the surfactant polysorbate 85 (HLB = 11) at different ratios, followed by drop-wise addition of water to attain a final extract content of 5% (*w*/*w*) [62]. With the exception of the extract-to-surfactant ratio at 9:1 (highly viscous), the JPE-NE prepared with all the other ratios (8:2, 7:3, 6:4, and 5:5) exhibited a high stability for seven days with a particle size, a PDI, and a ZP ranging from 164.4–221.8 nm, 0.170–0.266, and (−3.58)–(−8.81) mV, respectively. However, the JPE-NE was quite unstable at higher temperatures with the particle size increasing to 303.6 nm at 45 °C and 536 nm at 65 °C. The study also demonstrated a successful scale-up of JPE-NE preparation up to 25-fold while retaining good stability for seven days. For prolonged stability, the ZP should be further decreased to <−30 mV through modification of the nanoemulsion composition.

For encapsulation of anthocyanins from acai berry, Rabelo et al. [63] prepared a W/O nanoemulsion (ABE-NE) by mixing the oil phase containing 5% CR-310 emulsifier in medium-chain triglyceride (MCT) oil with the aqueous phase prepared with different weight fractions (10–30%) of acai berry extract, followed by homogenizing at 10,000 rpm for 5 min and passing four times through a high pressure homogenizer at 100 MPa. Compared to the MCT oil-dispersed ABE and blank-NE, the interfacial tension declined for ABE-NE with no significant change in density or viscosity. However, the incorporation of ABE into the nanoemulsion substantially decreased the particle size from 304.9–408.6 nm to 131.5–195.3 nm, which may have been due to the stabilizing effect of the high amino acid level found in acai berry. Likewise, upon storage at 4 °C for 30 days under dark, the particle size and the PDI for blank-NE increased respectively from 146.8 nm and 0.2 to 814.8 nm and 0.6, while lower values (<200 nm and <0.4) were found for NE loaded with 5% ABE. More than 70% of the total polyphenolic content was retained after 30-day storage, with the highest anthocyanin retention (95%) and the longest half-life (365 days) being shown for the 2% ABE-loaded NE containing 10% aqueous phase [63]. The enhanced stability of anthocyanin after incorporation into nanoemulsion may be accounted for by self-association or intermolecular copigmentation with aromatic groups and/or metal complexation.

## 6. Nanoliposome-Based Stability and Bioavailability of Anthocyanins

Nanoliposomes are spherical, single- or multi-layered lipid vesicles (unilamellar and multilamellar) formed through hydrophobic, hydrophilic, and van der Waals interactions when phospholipids are dispersed in water [28,34]. Due to their non-toxic, non-immunogenic, biocompatible, biodegradable, and amphiphilic nature, nanoliposomes have emerged as a potential delivery system for unstable bioactive compounds with poor bioavailability [36]. Although nanoliposomes protect the incorporated compounds within the aqueous inner core or the bilayer membrane and facilitate their release at a specific target site, liposomes can be rapidly destabilized due to flexible and fragile bilayer membranes accompanied by oxidation of unsaturated fatty acids [28]. However, through incorporation of antioxidant compounds such as anthocyanins as well as layer-by-layer coating with biopolymers, the nanoliposomes can be stabilized. Conventional preparation methods of nanoliposomes, including thin film hydration, ethanol injection, reverse phase evaporation, detergent removal, and dehydration-rehydration, are associated with some disadvantages, such as heterogeneous size distribution, low encapsulation efficiency, high energy cost with multistep operation, lack of long-term stability and reproducibility, and presence of solvent/surfactant residue [34,35]. To overcome these problems, some improved methods were developed, such as membrane contactor technology, freeze drying double emulsion, microfluidic hydrodynamic focusing, dual asymmetric centrifugation, cross-flow filtration detergent depletion, and supercritical CO_2_ technology [64,65]. In the following section, several reported nanoliposomes for enhancing the stability, the bioavailability, and the bioactivity of anthocyanins are discussed.

### 6.1. Nanoliposome with Standards

To establish a direct relationship between anthocyanin compounds and bioactivity, several authors used anthocyanin standards for preparation of nanoliposomes and demonstrated their efficiency in the treatment of diabetes mellitus [66] and cataract [67] as well as growth inhibition of Caco-2 cancer cells [68]. Gharib et al. [66] synthesized nanoliposomes with cyanidin chloride (Cy-NL) and delphinidin chloride (Dp-NL) by mixing a dried lipid layer consisting of soy lecithin and cholesterol (molar ratio 6:1) with 150 mg/mL of cyanidin or delphinidin chloride in phosphate buffered saline (PBS), sonicating at 4 °C, and extruding through a 100-nm polycarbonate membrane filter (12 passes). The in vitro studies showed that Cy-NL and Dp-NL at 100 mg/mL separately could reduce the rate of albumin glycation respectively by 85.4% and 91.5%, which was higher than those by Cy and Dp standards (54.0% and 69.5%). Likewise, an intravenous daily administration of 100 mg/kg of nanoliposomes or standards to streptozotocin (50 mg/kg)-induced diabetic mice for eight weeks could decrease the total cholesterol, the rate of albumin glycation, and the hemoglobin A1c (HbA1c) glycation, as well as the elevate glycogen level, with nanoliposomes being more effective than standards. Moreover, both Dp and Dp-NL exhibited a more pronounced effect both in vitro and in vivo studies compared to Cy and Cy-NL, which may have been due to the presence of one additional hydroxyl group in Dp and higher EE of Dp in Dp-NL (89%) compared to that for Cy in Cy-NL (85%), both of which are responsible for greater interaction with lipids in nanoliposomes [66].

In a later study, Zhang et al. [67] reported an increased precorneal residence time and enhanced deeper transport into corneal epithelium in a gamma-scintigraphy study using a rabbit eye model as well as the mitigation of selenite-induced oxidative stress in rats by N-trimethyl chitosan-coated Cy3G nanoliposomes (TMC-Cy3G-NL) prepared using lecithin and cholesterol by a reverse-phase evaporation method. The sphere-shaped TMC-Cy3G-NL with a particle size of 158.3 nm, a ZP of 31.7 mV, and an EE of 53.7% showed a 3.3- and 1.7-fold increment in precorneal residence time when compared to radioactive ^99m^Tc solution and blank nanoliposome in ^99m^Tc solution, respectively. Also, the treatment of the rabbit corneal epithelium sections with TMC-Cy3G-NL loaded with rhodamine-B dye for 150 min followed by monitoring with fluorescence showed a deeper penetration up to 40 μm into the corneal epithelium. In an antioxidative study, compared to uncoated Cy3G-NL, the TMC-Cy3G-NL was shown to prevent lipid peroxidation to a greater extent through the enhancement of superoxide dismutase and catalase activities as well as the reversal of reduced glutathione activity in sodium selenite-induced oxidative stress in the eye lens of Sprague-Dawley (SD) rats [67]. It is worth pointing out that TMC is one of the water-soluble derivatives of chitosan mainly used to increase the positive charge on nanoliposomes as well as enhance water-solubility over a wide pH range, thereby facilitating their preferential interaction with a negatively-charged corneal surface in ocular delivery. In addition, the TMC can stabilize the nanoliposomes from membrane fusions and enable slow release of Cy3G via diffusion.

More recently, based on a response surface methodology optimization, Liang et al. [68] prepared Cy3G nanoliposomes (Cy3G-NL) by the reverse-phase evaporation method using Cy3G at 0.17 mg/mL, a phosphatidylcholine/cholesterol (PC/CH) ratio at 2.87, and a rotary evaporation temperature at 41.41 °C to demonstrate their inhibition efficiency toward the growth of Caco-2 colorectal cancer cells. The Caco-2 cells are widely used as an in vitro small intestine mucosa model for predicting drug absorption. Based on the results of several preliminary optimization studies, the particle size of Cy3G-NL (165.78 nm) decreased following a decline in both temperature and PC level, while the EE rose at a higher PC/CH ratio and Cy3G concentration. Also, the thermal treatment at different temperatures (4 °C–85 °C) for 30 min showed Cy3G-NL to be stable in the range of 4 °C–40 °C; however, a further raise in temperature to 65 °C and 85 °C resulted in a 40.5% and a 65% loss of Cy3G, respectively. On the other hand, no significant change in EE and a 15% increase in particle size was observed upon Cy3G-NL storage at 4 °C for 21 days. Given the possible degradation of Cy3G-NL in the stomach and the intestine, the in vitro release of Cy3G in simulated gastric fluid (SGF) and simulated intestinal fluid (SIF) monitored for 4 h showed a 18.6% and a 35.6% release from Cy3G-NL, respectively (Figure 4A-1). Furthermore, following a rise in Cy3G-NL concentration from 0.5 to 0.25 mg/L, the Caco-2 cells became abnormal in shape (irregular cellular shrinkage), and the cell viability decreased from 90.11% to 32.56% in a dose-dependent manner with the IC_50_ value being 0.19 mg/mL (Figure 4A-2) [68].

### 6.2. Nanoliposome with Natural Extracts

Besides the anthocyanin standards-based nanoliposomes, preparation and application of nanoliposomes with anthocyanin-rich natural extracts were also studied to explore the advantages of stability and synergistic bioactivity through self-association of several anthocyanin compounds and their copigmentation with other organic acids and functional compounds.

In an attempt to enhance the anthocyanin stability and evaluate the melanogenic activity, Hwang et al. [72] prepared multilamellar nanoliposomes from anthocyanin extracts of *Hibiscus sabdariffa* Linn. (HS-NL) using lecithin and cholesterol. The HS-NL with an EE of 55% was found to remain unchanged in particle size with increasing storage time but significantly reduced with a raise in temperature from 4 °C (206.2 nm) to 60 °C (157.5 nm). However, during storage, about 35–40% of anthocyanin was shown to release from HS-NL into PBS at 37 °C after 8 h and rose gradually to 45% after 24 h. Upon incorporation of anthocyanin extract at 20 and 50 mg/mL into nanoliposome, the DPPH radical scavenging activity increased from 11% to 64% for the former and 12% to 76% for the latter. Likewise, at the same dose (5, 10, 20, and 50 mg/mL), the HS-NL showed a higher inhibition (23, 35, 43, and 60%) on melanin production in human A375 melanoma cells than anthocyanin extract (8, 14, 23, and 30%). The significant reduction in melanin synthesis was mainly due to a dose-dependent inhibition of tyrosinase activity and protein expression associated with tyrosinase and melanocyte inducing transcription factor (MITF) in A375 melanoma cells [72].

Multilayered liposomes containing more than a single bilayer membrane were also prepared, and their efficiency in stabilizing anthocyanins was studied. These multilamellar vesicles possess the ability to withstand harsh conditions existing in the stomach and GI tract when compared to unilamellar vesicle-based single-layered liposomes. A variety of polysaccharides such as chitosan and/or pectin can be used as protective coating in multilamellar liposomes by the layer-by-layer technique. Gibis et al. [69] prepared a primary nanoliposome (HS-NL) using soy lecithin and anthocyanin extract from *H. sabdariffa* (HS) by high pressure homogenization at 22,500 psi (five passes) as well as a single-layered liposome with chitosan (HS-CNL) and a double-layered liposome with chitosan followed by pectin (HS-CPNL). Figure 4B shows the schematic representation of the step-wise preparation method involved in the formation of multilayered nanoliposomes. Incorporation of HS extract into liposomes did not cause any significant change in particle size compared to blank liposomes without anthocyanin extract. Upon coating HS-NL with chitosan and chitosan/pectin, the particle size increased respectively from 46 nm to 65 nm and 200 nm accompanied by a shift in surface charge from −26 mV to 70 mV and −20 mV, while the duration of stability decreased from 145 days (HS-NL) to 30 days (HS-CPNL) based on the level of hexanal released measured by using gas chromatography with flame ionization detection. For coated liposomes (HS-CNL and HS-CPNL), following a rise in the number of coatings, coating percentage and HS extract concentration, the surface coverage, the particle size, the distribution, and the PDI increased. Consequently, the multilayered liposomes (HS-CPNL) with the lowest concentration of HS anthocyanin extract were shown to provide the highest stability over a 30-day period and thus can be an effective competent carrier system for anthocyanins [69].

In another study, Gultekin-Ozguven et al. [73] prepared lecithin-based chitosan-coated nanoliposomes (CH-NL) by both high shear disperser (9500 rpm for 10 min) and high pressure homogenizer (25,000 psi, five passes) to stabilize and enhance the bioaccessibility of anthocyanin-rich black mulberry extract (BME). Upon coating with chitosan, the particle size of liposomes with BME was shown to increase from 173 to 473 nm, while the surface charge shifted from negative to positive with the ZP values ranging from 39.94–41.87 mV. Furthermore, with an EE of 76.8% for 0.2% BME and 0.4% *w*/*v* chitosan coating, the in vitro anthocyanin bioaccessibility was enhanced compared to the uncoated BME-NL. Also, the incorporation of CH-BME-NL into dark chocolates could minimize loss of anthocyanins compared to spray-dried BME prepared at different pH levels (4.5, 6.0, and 7.5) and temperatures (40, 60, and 80 °C).

Lecithins obtained from different food sources (egg yolk, soybean, and sunflower) were used for the preparation of nanoliposomes with elderberry extract by the thin lipid film hydration technique and evaluation of their stability by measuring particle size, surface charge, structure, and EE [74]. Of the three lecithins tested, soybean lecithin was found to exhibit the highest stability, as shown by ZP (−36.4 mV), small particle size (205 nm), and low PDI (0.49). Although a relatively higher EE was obtained for liposomes prepared using egg yolk lecithin (48%) and sunflower lecithin (68%), soybean lecithin-based liposomes (25%) possessed more uniform size and structure (highest homogeneity) compared to the other two liposomes. Also, the stability was enhanced upon storage at 4 °C in the dark with particle size and PDI decreasing to 166 nm and 0.36 after four weeks, respectively. Moreover, the larger particle size (>100 nm) for all three liposomes could be attributed to the presence of at least two bilayers, while the smallest particle size with high homogeneity for soybean-based liposomes may have been due to low phosphatidylcholine and high phosphatidic acid levels containing less amino acid units.

Accordingly, ascorbic acid (AA) can be added to fruit juices to prevent enzymatic browning reactions and enhance nutritional properties. However, the AA can affect the stability of anthocyanins and cause degradation, resulting in nutritional loss. To prevent the anthocyanin degradation by AA, Guldiken et al. [70] prepared nanoliposomes with different levels of anthocyanin-rich black carrot extract (0.1, 0.2, and 0.4%) (BCE-NL) and soy lecthin (1, 2, and 4%), with particle sizes, PDIs, ZPs, and EEs ranging from 41–46 nm, 0.274–0.342, (−18.9)–(−23.2), and 32–50%, respectively. Furthermore, the EE rose following a decline in BCE level and an increase in lecithin concentration. Upon the addition of AA (0.01, 0.025, 0.05, and 0.1%), a dose- and time-dependent decrease in color and stability was observed for both BCE and BCE-NL after 24 h storage. However, the anthocyanin degradation rate was substantially reduced for BCE-NL (Figure 4C-1). The anthocyanin degradation may have also been caused by the oxidation of flavylium cations by several degradation products of AA, such as dehydroascorbic acid, furfurals, and hydrogen peroxide. However, the vesicle bilayer in liposomes may have acted as a shield between AA and BCE, thereby protecting anthocyanin from degradation (Figure 4C-2). More recently, the same authors demonstrated that the spray-dried and the chitosan-coated BCE-NL with particle size at 82.7 nm could increase both the physical and the chemical stability of anthocyanins [75].

Rafiee et al. [71] also compared different levels of soy lecithin (1, 2, and 3%) and extract (500, 750, and 1000 ppm) for preparation of nanoliposomes with pistachio green hull extract (PHE-NL) and evaluation of anthocyanin stability. PHE is a rich source of phenolic compounds containing anthocyanins at 118.6 mg Cy3G/g. Both unilamellar and spherical-shaped nanoliposomes were prepared by the thin hydration method with PS ranging from 90.4–103.8 nm, PDI from 0.069–0.123, ZP from (−40.2)–(−51.5) mV, and EE from 26–53% (Figure 4D). Compared to the PHE and unloaded nanoliposomes, the PHE-loaded nanoliposomes could increase both the phase transition temperature and the oxidation onset temperature, implying that a high thermal and lipid oxidation stability was attained through polyphenol loading. Also, only a minor change in particle size, PDI, and ZP was observed upon storage at 4 °C for two months, while 75% of the encapsulated polyphenols was retained.

### 6.3. Nanoliposomes by Supercritical Carbon Dioxide Method

Although conventional methods as described above can be used for the preparation of nanoliposomes, several disadvantages do exist, which include high energy cost, heterogeneous size distribution, organic solvent/surfactant residue, long term instability, non-reproducibility, and low entrapment of hydrophilic compounds [34,35]. Several recent techniques have significantly reduced heterogeneity and particle size while simultaneously improving EE [65]. However, the use of organic solvents and surfactants in these techniques still remains a challenge. Supercritical carbon dioxide (SCD) technology provides a promising alternative to prepare liposomes for encapsulation of both hydrophobic and hydrophilic compounds [64]. The SCD being a dense supercritical fluid offers a solvating power similar to liquid organic solvents and can be operated under mild conditions just above the critical temperature of CO_2_ (31.1 °C, 74 bar) [76]. Also, it is non-toxic and eco-friendly, possessing several advantages including, high diffusivity, low interfacial tension, tunable density, and low viscosity [64]. The SCD technology can be conveniently used for encapsulating thermolabile compounds and can be scaled-up for industrial production. However, the capital cost is high.

**Figure 5:**
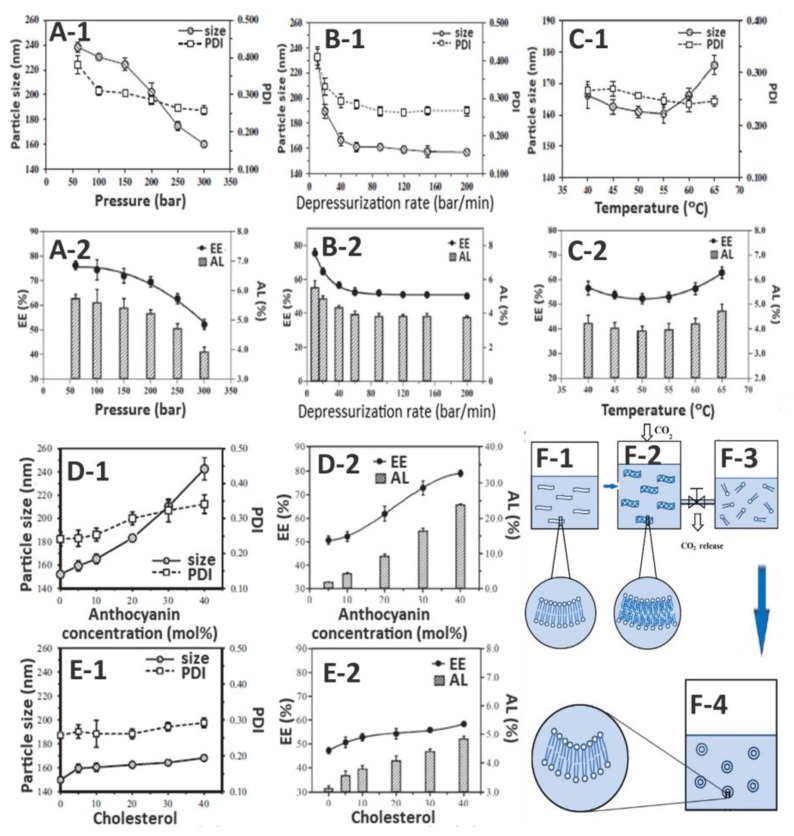
Anthocyanin nanoliposome characteristics as affected by several parameters involved in the supercritical carbon dioxide (SCD) preparation method and the mechanism of nanoliposome formation. Panels A-E show the effects of different SCD pressure (**A**), depressurization rate (**B**), and temperature (**C**), as well as anthocyanin (**D**) and cholesterol (**E**) concentration on particle size/polydispersity index (PDI) (**A1,B1,C-1,D-1,E1**) and encapsulation efficiency (EE)/anthocyanin loading (AL) (**A-2,B-2,C-2,D-2,E-2**). Panel F shows the schematic mechanism of nanoliposome formation by the SCD method, depicting the normal phospholipid curvatures at ambient condition (**F1**), expanded phospholipid bilayers after pressurization and equilibration with CO_2_ (**F2**), instantaneous dispersion of discrete phospholipid molecules during depressurization and CO_2_ release (**F3**), and formation of nanoliposome vesicles through hydrophobic interactions after depressurization (**F4**) (adapted with permission from references [64,65,76]).

Zhao and Temelli [65] prepared bilberry anthocyanin-loaded nanoliposomes (BA-NL) by a single step supercritical carbon dioxide process through the optimization of different pressure (P, 60–300 bar), depressurization rate (DPR, 10–200 bar/min), and temperature (T, 40–65 °C). Initially, a crude suspension was prepared by mixing 10% bilberry anthocyanin extract with 1.33% soy lecithin and 10% cholesterol (Figure 4E). It was found that an elevation in P and DPR generated smaller particles with higher homogeneity (Figure 5A-1,B-1,C-1), while a rise in T affected spherical morphology. However, at higher P and DPR, both EE and anthocyanin loading efficiency significantly decreased (Figure 5A-2,B-2,C-2). The spherical BA-NL with a particle size of 160 nm, a PDI of 0.26, a ZP of -44.3 mV, an EE of 52.2%, and an anthocyanin loading of 3.8% could be prepared by using the optimum condition with P at 300 bar, DPR at 90 bar/min, and T at 50 °C (Figure 5A–C). Compared with the conventional thin film hydration method (TFH), the SCD method could provide nanoliposomes with smaller particle size, higher homogeneity, and greater stability (SCD for three weeks, TFH for less than one week), all of which may have been attributed to the cooling effect caused by the Joules-Thomson effect during depressurization [65].

In a later study, Zhao et al. [76] prepared BA-NL by the same SCD method shown above and investigated their characteristics as affected by varying anthocyanin (0–40%) (Figure 5D) and cholesterol (0–40%) (Figure 5E) levels. Adopting optimized SCD parameters as described above, a rise in anthocyanin and cholesterol concentration could raise both EE and anthocyanin loading, accompanied by a significant increase of both particle size and PDI, with cholesterol showing a less pronounced effect than anthocyanin (Figure 5D-1,E-1). In addition, a higher anthocyanin and cholesterol level could elevate both EE and anthocyanin loading (Figure 5D-2,E-2) as well as enhance asymmetry in particle shapes. An in vitro release study revealed a slow anothocyanin release in SGF, whereas a rapid release due to vesicle degradation by pancreatin was found in SIF, suggesting a need for multilamellar fabrication or coating of nanoliposomes with polymers. Thus, with an optimized anthocyanin level at 10% and cholesterol at 20%, the BA-NL with a particle size of 159 nm, a PDI of 0.244, a ZP of −40.2 mV, and an EE of 50.6% could be successfully prepared. A schematic mechanism of nanoliposome formation by the SCD method is shown in Figure 5F [76].

## 7. Conclusions and Future Perspectives

This review presents an overview of the recent nanotechnological strategies for the enhancement of anthocyanin stability and bioavailability. Based on published reports, it is evident that both the preparation and the application of nanoemulsion/nanoliposome systems for enhancing anthocyanin stability are still in a preliminary stage. The future studies should focus on preparing nanoemulsions and nanoliposomes possessing high encapsulation efficiency (>80%) with particle size <100 nm and surface charge <−30 or >+30 mV. Also, their stability needs to be enhanced by single or multilayered coating with different natural biopolymers or proteins/polysaccharides through optimization of coating material dose and maintenance of small particle size. Anthocyanin extracts obtained from a wide variety of natural sources should be used for nanoemulsion/nanoliposome preparation, and their efficiency should be compared with anthocyanin standards for possible elucidation of synergistic effects. As the current literature is dominated with W/O nanoemulsion preparations, studies involving O/W nanoemulsion should be increased due to their wide applicability in the biomedical field. For nanoliposomes, phytosterols can be substituted for cholesterol to minimize undesirable health effects, and the application of the supercritical CO_2_ method should be improved for possible large-scale industrial application. Instead of limiting to few preparation methods and evaluating only the physicochemical stability, different low and high energy methods or their combinations should be employed to prepare nanoemulsion and nanoliposomes for evaluation of biological activity both in vitro and in vivo for possible future clinical application.

## Figures and Tables

**Table 1:** Total content of anthocyanins in common fruits and vegetables in the United States (adapted with permission from reference [6]).

Food Variety	Total Anthocyanin (mg/100 g FW) ^1^	Food Variety	Total Anthocyanin (mg/100 g FW) ^1^
Fruits Apple Fuji (*n* = 4) Gala (*n* = 3) Red delicious (*n*-4) Blackberry Blackberry (*n* = 4) Marion blackberry (*n* = 1) Blueberry Cultivated (*n* = 7) Wild (*n* = 1) Cherry (sweet) Chokeberry (*n* = 1) Cranberry (*n* = 3) Currant Black currant (*n* = 6) Red currant (*n* = 1) Elderberry (*n* = 1) Gooseberry Batch 1 (*n* = 2) Batch 2 (*n* = 1) Batch 3 (*n* = 1)	1.3 ± 0.7 2.3 ± 0.8 12.3 ± 1.9 245 ± 68 300.5 386.6 ± 77.7 486.5 122 ± 21.3 1480 140 ± 28.5 476 ± 115 12.8 1375 10.4 ± 0.1 2.2 0.7	10. Grape Red grape (*n* = 5) Concord grape (*n* = 1) 11. Nectarine (*n* = 7) 12. Peach (*n* = 8) 13. Plum Plum (*n* = 8) Black plum (*n* = 2) 14. Raspberry Black raspberry Red raspberry 15. Strawberry Strawberry (*n* = 8) Strawberry OSC ^2^ (*n* = 1) Vegetables Black bean (*n* = 1) Eggplant (*n* = 1) Red cabbage (*n* = 4) Red leaf lettuce (*n* = 8) Red onion (*n* = 1) Red radish (*n* = 9) Small red bean (*n* = 1)	26.7 ± 10.9 120.1 6.8 ± 1.5 4.8 ± 1.2 19.0 ± 4.4 124.5 ± 21.6 687 92.1 ± 19.7 21.2 ± 3.3 41.7 44.5 85.7 322 ± 40.8 2.2 ± 1.5 48.5 100.1 ± 30.0 6.7

^1^ The anthocyanin contents are determined by high-performance liquid chromatography–diode array detection–electrospray ionization–tandem mass spectrometry (HPLC-DAD-ESI-MS/MS) and expressed on fresh weight (FW) basis, with single value indicating analysis of one sample (*n* = 1) and mean ± standard deviation values denoting analyses of multiple samples (*n* ≥ 2). ^2^ Oregon Strawberry Commission.

## Abbreviations

AA	ascorbic acid
ABE	acai berry extract
ABE-NE	acai berry extract nanoemulsion
ACT	anthocyanin acyltransferase
AFM	atomic force microscopy
AL	anthocyanin loading
ANCs	anthocyanins
ANS	anthocyanidin synthase
BAE	blueberry anthocyanin extract
BAE-ME	blueberry anthocyanin extract microemulsion
BA-NL	bilberry anthocyanin extract nanoliposome
BCE	black carrot extract
BCE-NL	black carrot extract nanoliposome
BHT	butylated hydroxytoluene
BME	black mulberry extract
BME-NL	black mulberry extract nanoliposome
BPAE-NE	blueberry pomace anthocyanin extract nanoemulsion
CB	anthocyanin-rich cranberry
CB/GTC-NE	cranberry/green tea catechin extract nanoemulsion
CH-BME-NL	chitosan black mulberry extract nanoliposome
CHI	chalcone isomerase
CHS	chalcone synthase
Cy	cyanidin
Cy3G	cyanidin-3-glucoside
Cy3G-NL	cyanidin-3-glucoside nanoliposome
Cy-NL	cyanidin nanoliposome
DFR	dihydroflavonol-4-reductase
Dp	delphinidin
Dp-NL	delphinidin nanoliposome
DPPH	1,1-diphenyl-2-picrylhydrazyl
DPR	depressurization rate
EE	encapsulation efficiency
F3′,5′H	flavonoid 3′,5′-hydroxylase
F3′H	flavonoid 3′-hydroxylase
F3H	flavonoid 3-hydroxylase
FW	fresh weight basis
GI	gastrointestinal
GTC	green tea catechin extract
HbA1c	hemoglobin A1c
HEM	high energy method
HLB	hydrophilic-lipophilic balance
HPLC-DAD-ESI-MS/MS	high-performance liquid chromatography-diode array detection-electrospray ionization-tandem mass spectrometry
HS-CNL	chitosan coated *Hibiscus sabdariffa* nanoliposome
HS-CPNL	chitosan/pectin coated *Hibiscus sabdariffa* nanoliposome
HS-NL	Hibiscus sabdariffa nanoliposome
IC_50_	half-maximal inhibitory concentration
JPE-NE	jaboticaba peel extract nanoemulsion
LEM	low energy method
Malonyl-CoA	malonyl-coenzyme A
MCT	medium chain triglyceride
ME	microemulsion
MITF	melanocyte inducing transcription factor
MPE-NE	mangosteen peel extract nanoemulsion
Mv	malvidin
NE	nanoemulsion
O/W	oil-in-water
OMT	O-methyl transferase
OSC	Oregon strawberry commission
P	pressure
PC/CH	phosphatidylcholine/cholesterol
p-Coumaroyl-CoA	p-coumaroyl-coenzyme A
PDI	polydispersity index
PEG 400	polyethylene glycol 400
Pg	pelargonidin
PHE-NL	pistachio hull extract nanoliposome
Pn	peonidin
Pt	petunidin
RCE	red cabbage extract
RCE-SLNs	red cabbage extract solid lipid nanoparticles
SCD	supercritical carbon dioxide
SD	Sprague-Dawley rats
SGF	simulated gastric fluid
SIF	simulated intestinal fluid
SLNs	solid lipid nanoparticles
SNEDDS	self-nanoemulsifying drug delivery system
SPE-ME	purple sweet potato extract microemulsion
T	temperature
^99m^Tc-CB/GTC-NE	metastable Technetium-99 isomer radiolabeled Cranberry/green tea catechin extract nanoemulsion
TEM	transmission electron microscopy
TFH	thin film hydration
TMC	N-trimethyl chitosan
TMC-Cy3G-NL	N-trimethyl chitosan coated cyanidin-3-glucoside nanoliposome
UFGT	uridine diphosphate-sugar flavonoid 3-O-glycosyltransferase
UV	ultraviolet
W/O	water-in-oil
W/O/W, W_1_/O/W_2_	water-in-oil-in-water double emulsion
w/w	weight per weight
WPI	whey protein isolate
ZP	zeta potential
